# The Association of Lone-Motherhood with Smoking Cessation and Relapse: Prospective Results from an Australian National Study

**DOI:** 10.3390/ijerph10072906

**Published:** 2013-07-12

**Authors:** Mohammad Siahpush, Raees A. Shaikh, Melissa Tibbits, Terry T-K Huang, Gopal K. Singh

**Affiliations:** 1 Department of Health Promotion, Social and Behavioral Health, College of Public Health, University of Nebraska Medical Center, 984365 Nebraska Medical Center, Omaha, NE 68198-4365, USA; E-Mails: raees.shaikh@unmc.edu (R.A.S.); mtibbits@unmc.edu (M.T.); tthuang@unmc.edu (T.T.-K.H.); 2 Office of Epidemiology and Research, Division of Epidemiology, HRSA/Maternal and Child Health Bureau, U.S. Department of Health & Human Services, 5600 Fishers lane, Room 18-41, Rockville, MD 20857, USA; E-Mail: gsingh@hrsa.gov

**Keywords:** lone-motherhood, smoking cessation, relapse

## Abstract

The aims were to examine the association of lone-motherhood with smoking cessation and relapse, and to investigate the extent to which this association was accounted for by socioeconomic status (education, occupation, and income), social support, and mental health. We used data from 10 yearly waves (2001 to 2010) of the Household Income and Labour Dynamics in Australia (HILDA) survey. Response rate in the first wave was 66%. Logistic regression was used to examine the effect of lone-motherhood and other covariates on smoking cessation (n = 2,878) and relapse (n = 3,242). Results showed that the age-adjusted odds of smoking cessation were 32% smaller among lone mothers than partnered mothers (*p* = 0.004). The age-adjusted odds of relapse was 172% greater among lone mothers than partnered mothers (*p* < 0.001). We found that socioeconomic status, social support, and mental health account for some of the association of lone motherhood and cessation and relapse. While efforts to reduce the smoking prevalence among lone mothers should focus on their material deprivation, availability of social support, and addressing mental health issues, other factors unique to the lives of lone mothers also need to be taken into account. More research is needed to discover other factors that can explain the association of lone-motherhood and smoking behavior.

## 1. Introduction

Lone mothers are among the most socially disadvantaged groups in many countries [1,2,3,4,5,6] and suffer from a notably higher prevalence of smoking than partnered mothers and other women [2,3,7,8,9,10,11]. In Australia, smoking prevalence among lone mothers, who comprise 12% of all families, is more than three times that of partnered mothers [3]. Australian studies have shown that the association between lone motherhood and higher smoking prevalence is partly due to lone mothers’ lower socioeconomic status, higher prevalence of mental health issues, larger number of friends who smoke, and lower age of smoking initiation compared to other women [2,3]. We know of no studies that compare smoking cessation and relapse between lone mothers and partnered mothers. Our purpose was to examine this association and the factors that accounted for it, in a national sample in Australia. We examined three such factors: socioeconomic status, social support, and mental health.

First, socioeconomic status (SES) is known to be strongly associated with both lone motherhood and smoking. About half of lone mothers in Australia have no paid work [12] and those who are employed are more likely than partnered mothers to face employment instability, ongoing financial strain, and poverty [13,14]. Lone mothers, compared to other women are more likely to have lower levels of education and income, reside in rented housing, and live in lower socioeconomic areas [2]. Lower socioeconomic status is also a known predictor of smoking cessation and relapse. For example, smokers with lower levels of income or education are less likely to make a quit attempt and stay abstinent [15]. Similarly, smokers with a lower occupational position are more likely to relapse after a quit attempt [16]. Additionally, when compared to those who do not experience financial strain, smokers with financial strain are less likely to quit smoking and ex-smokers with financial strain are more likely to relapse [17].

Second, there is evidence that social support is associated with both lone motherhood and smoking. In Australia, lone mothers are notably less likely than partnered mothers to report having social support, measured using a scale that includes items such as having a lot of friends or friends that help when needed [1]. Similarly, lone mothers in the US are reported to be four times more likely than partnered mothers to have contact with friends fewer than twice a month [1,7]. While some studies have shown that partner support and general social support are associated with lower smoking prevalence and a higher likelihood of smoking cessation [18,19,20], other studies provide little evidence that social support facilitates smoking cessation [21].

Third, mental health has also been found to be associated with both lone motherhood and smoking. An Australian study reported that lone mothers are at a much greater risk of moderate or severe mental disability than partnered mothers [1]. Another Australian study found that compared to all other women, lone mothers are more likely to report poor mental health [2]. Similarly, research conducted in other countries has reported a greater risk of depression, anxiety, or psychological distress among lone mothers than other women [22,23,24]. Mental health is also a predictor of smoking behavior. Smokers with anxiety disorder are less likely to quit smoking than others [25]. Having a history of depression is associated with greater withdrawal symptoms among individuals who have recently quit smoking [26]. Smokers with depressive symptoms are also more likely to relapse after a quit attempt than others [27]. 

Our aim was to use nationally representative data and compare cessation and relapse rates between lone mothers and partnered mothers in Australia. We also aimed to examine the extent to which the effect of lone motherhood on smoking cessation and relapse was accounted for by socioeconomic status, social support, and mental health.

## 2. Methods

### 2.1. Data

We used data from Waves 1 (2001) to 10 (2010) of the Household Income and Labour Dynamics in Australia (HILDA) yearly survey, which is a national longitudinal study based on a multi-stage area sample of households. HILDA includes a face-to-face interview and a self-completion questionnaire about income, employment, health, and wellbeing. Data collection in Wave 1 was conducted in 2001 with an overall response rate of 66%. Overall attrition from Wave 1 to 2 was 13% and averaged about 5% in subsequent waves. Sample size in each wave was about 18,000 individuals. The number of observations in the ten waves was a total of 177,938. About 51% of the sample was women in each wave. The prevalence of current smoking generally declined from the first to the tenth wave and ranged between 19.8% and 24.1%. Women, who were less than 15 years of age, as well as those women who in two consecutive waves of the survey did not have at least one child under the age of 15, were excluded from the study. Methodological details about HILDA are described elsewhere [28]. 

### 2.2. Measurement of Outcomes

The outcomes were smoking cessation and relapse. In Wave 1, respondents were asked: “Do you smoke cigarettes or any other tobacco products?” They were provided with three options, “No, I have never smoked”, “No, I have given up smoking”, and “Yes”, identifying never-smokers, ex-smokers, and current smokers, respectively. In Wave 2 and subsequent waves, respondents were asked the same smoking question with the following response options: “No, I have never smoked”, “No, I no longer smoke”, “Yes, I smoke daily”, “Yes, I smoke at least weekly (but not daily)”, and “Yes, I smoke less often than weekly”. The first and second options define never-smokers and ex-smokers, respectively. The last three options define current smokers. In the cessation analysis, respondents who were a current smoker in a given wave and reported to be an ex-smoker in a subsequent wave were considered to have quit smoking and were compared with respondents who reported to be a current smoker in two consecutive waves (*i.e.*, continued smokers). In the relapse analysis, respondents who were an ex-smoker in a given wave and reported to be a current smoker in a subsequent wave were considered to have relapsed and were compared with respondents who were an ex-smoker in two consecutive waves (*i.e.*, those who did not relapse).

### 2.3. Measurement of Covariates

We defined lone-mothers as women who in two consecutive waves of the survey did not have a partner and had at least one child under the age of 15 years in the household. We defined partnered mothers as women who in two consecutive waves of the survey were married or in a de facto relationship and had at least one child under the age of 15 years in the household [2,3]. A “partner” for a married woman was the spouse with whom she was living. For the women who were never married, separated, divorced or widowed, a “partner” was someone with whom they were living in a relationship. For each instance where a respondent was a lone mother or a partnered mother in two consecutive waves, we assessed her quit or relapse status using data from those two waves. If she was a smoker in the first wave, she was included in the cessation analysis and was considered to have quit if she reported to be an ex-smoker in the second wave. On the other hand, if she was an ex-smoker in the first wave she was included in the relapse analysis, and was considered to have relapsed if she reported to be a smoker in the second wave. All covariates were measured in the first of the two consecutive waves that an individual was either a lone mother or a partnered mother. Because we utilized data from 10 survey waves, many individuals were a lone mother or a partnered mother in two consecutive waves more than once in the duration of the study and thus contributed more than one observation to the analysis.

Two indicators of socioeconomic status were employed. Education was categorized into three groups: year 12 (final year of high school) or below; trade certificate or diploma; and university degree [29]. HILDA provides imputed values of household income for instances of survey nonresponse. The imputations are based on the nearest neighbor regression, population carryover, and Little and Su methods [28]. The nearest neighbor regression technique imputes missing values based on the predicted values from a cross-sectional regression model for the variable to be imputed [30]. The population carryover method imputes missing values by utilizing the responding information from observations from surrounding waves [31]. The Little and Su method imputes missing values using information on trend across waves and departure from the trend in the waves where the income component has been reported [32]. We divided income into quintiles.

Mental health was created as a summary measure using items from the four sub-scales of the “Medical Outcomes Study 36-item Short-Form Health Survey”, also known as SF-36 questionnaire: vitality, role limitation due to emotional problems, emotional-wellbeing, and social functioning [33]. These four scales together make up the mental health component of SF-36 and include items related to such concepts as “feeling so down in the dumps, nothing could cheer you up”, “physical/emotional problems interfering with social activities”, “been a nervous person”, and “feeling worn out”. We combined the scores for all the items from the four sub-scales and standardized this combined measure to create the mental health scale, with higher scores indicating better mental health. The Cronbach’s alpha reliability coefficient for this scale was 0.99 and 0.82 in the cessation and relapse analysis, respectively. 

Perceived social support was measured using 10 questionnaire items such as: “I seem to have a lot of friends”, “There is someone who can always cheer me up when I’m down”, and “When I need someone to help me out, I can usually find someone” [1,34,35]. We created a scale based on the scores on these items with higher scores indicating greater social support. The Cronbach’s alpha reliability coefficient for this scale was 0.91 and 0.84 in the cessation and relapse analysis, respectively. 

We also considered number of dependent children in the household as control variable, but we did not include it in the models because it was not associated with the outcomes and did not alter the effect of other predictors.

### 2.4. Statistical Analysis

As the outcome variable in both cessation and relapse analysis was dichotomous, we used logistic regression to examine the effect of covariates. Cases with missing values for any of the study variables, which comprise 6.9% in the cessation analysis and 5.9% in the relapse analysis, were excluded from each analysis. The total number of observations in the cessation analysis was 2,878 representing 450 unique individuals across all survey waves. The total number of observations in the relapse analysis was 3,242 representing 432 unique individuals across all survey waves. For computing standard errors for regression estimates, we have adjusted for the clustered nature of the sampling design by using the linearized variance estimator [36]. In each table of regression results, five columns of odds ratios are presented representing five sequential models. The first model provides crude odds ratios; the second model adjusts for age; the third model adds socioeconomic variables, the fourth model adds social support; and finally the last model adds mental health. A reduction in the odds ratio for the effect of lone motherhood gives an indication of the extent to which its association with cessation and relapse is mediated by various factors. Stata SE version 12 was used in all analyses [37].

## 3. Results

Table 1 provides descriptive statistics for observations in the cessation and relapse analysis. In the cessation analysis, 33.5% of the observations were lone mothers who had a cessation rate of 10.3%, and 66.5% were partnered mothers with a cessation rate of 14.4%. Lower education, occupational position, income, social support, and mental health were associated with a lower cessation rate. In the relapse analysis, 14.4% of observations were lone mothers who had a relapse rate of 18.4%, and 85.6% were partnered mothers with a relapse rate of 7.9%. Lower education, income, social support and mental health were associated with a higher relapse rate.

Table 2 shows the association of lone motherhood with smoking cessation and the extent to which this association is explained by SES, social support, and mental health. The age-adjusted results indicate that the odds of cessation were 32% lower in lone mothers than partnered mothers (OR: 0.68; 95% CI: 0.51–0.91). This effect was attenuated to 30% (OR: 0.70; 95% CI: 0.50–0.98) when socioeconomic indicators were added to the model and to 29% when social support and mental health were added (OR: 0.71; 95% CI: 0.51–0.99). In the final model, higher level of education was associated with a higher likelihood of smoking cessation. Age, income, social support, and mental health were not associated with smoking cessation.

In the relapse analysis, about 14% of the sample consisted of lone mothers and 86% consisted of partnered mothers. Among lone mothers 18% relapsed, while among partnered mothers 8% did so. Table 3 shows the association of lone motherhood with relapse and how much of this association is accounted for by SES, social support, and mental health. The age-adjusted results indicate that the odds of relapse were 172% (OR: 2.72; 95% CI: 1.95–3.79) higher among lone mothers. This effect was reduced to 146% (OR: 2.46; 95% CI: 1.69–3.57) after adding SES to the model, to 136% (OR: 2.36; 95% CI: 1.61–3.46) after adding social support, and to 127% (OR: 2.27; 95% CI: 1.55–3.33) after adding mental health to the regression model. In the final model, higher age, education and better mental health were associated with lower probabilities of relapse. Income and social support did not have an association with relapse. 

**Table 1:** Characteristics of respondents in the cessation (n = 2,878^ a^) and relapse (n = 3,242^ b^) analysis.

Covariates	Cessation analysis		Relapse analysis	
	% in sample	% quit	% in sample	% relapse
*Lone/partnered mother*				
Lone mother	33.50	10.17	14.44	18.38
Partnered mother	66.50	14.37	85.56	7.86
*Age*				
Under 25	9.31	10.45	3.39	25.45
25–39	36.80	13.03	25.79	13.76
40–54	44.58	13.56	52.62	7.74
55+	9.31	12.31	18.20	4.92
*Education*				
High school or less	60.46	11.21	43.28	10.76
Diploma or certificate	28.67	14.18	29.70	10.49
University degree	10.88	19.49	27.02	5.94
*Income*				
First quintile (low income)	20.01	10.59	20.02	15.41
Second quintile	20.01	10.59	19.99	8.95
Third quintile	19.98	14.96	20.02	7.86
Fourth quintile	20.01	11.28	19.99	7.25
Firth quintile (high income)	19.98	17.39	19.99	7.41
*Social support* *^c^*				
Below median	50.52	12.65	51.54	10.77
At or above median	49.48	13.27	48.46	7.89
*Mental health* *^c^*				
Below median	51.95	12.11	50.12	11.08
At or above median	48.05	13.88	49.88	7.67

Note: ^a^ The observations in the cessation analysis represent 450 unique individuals. ^b^ The observations in the relapse analysis represent 431 unique individuals. ^c^ Higher social support and mental health scores indicate better mental health. In this table, the mental health and social support scales have been conveniently dichotomized. Data are from Household Income and Labour Dynamics in Australia (HILDA) survey (2001–2010).

**Table 2:** Odds ratios (95% CI) for the effect of lone-motherhood, socioeconomic status, social support, and mental health on the probability of smoking cessation (n = 2,878).

Covariates	Crude OR	Adjusted OR	Adjusted OR	Adjusted OR	Adjusted OR
*Lone/partnered mother*					
Lone mother	0.67 (0.51–0.90)	0.68 (0.51–0.91)	0.70 (0.50–0.98)	0.71 (0.51–0.99)	0.71 (0.51–0.99)
Partnered mother	1.00	1.00	1.00	1.00	1.00
*p*	0.007	0.006	0.037	0.046	0.047
*Age*					
Under 25	1.00	1.00	1.00	1.00	1.00
25–39	1.28 (0.80–2.05)	1.24 (0.77–1.99)	1.18 (0.73–1.92)	1.16 (0.72–1.88)	1.16 (0.72–1.88)
40–54	1.34 (0.84–2.15)	1.29 (0.81–2.07)	1.17 (0.72–1.89)	1.14 (0.70–1.85)	1.14 (0.70–1.85)
55+	1.20 (0.68–2.14)	1.21 (0.68–2.15)	1.03 (0.56–1.87)	1.01 (0.55–1.83)	1.01 (0.55–1.83)
*p*	0.644	0.759	0.851	0.869	0.870
*Education*					
High school or less	1.00		1.00	1.00	1.00
Diploma or certificate	1.31 (1.02–1.69)		1.31 (1.01–1.69)	1.31 (1.02–1.69)	1.31 (1.02–1.70)
University degree	1.92 (1.32–2.80)		1.87 (1.27–2.77)	1.87 (1.26–2.76)	1.87 (1.27–2.76)
*p*	0.001		0.002	0.002	0.002
*Income*					
First quintile (low income)	1.00		1.00	1.00	1.00
Second quintile	1.00 (0.68–1.48)		0.86 (0.57–1.28)	0.85 (0.57–1.28)	0.85 (0.57–1.28)
Third quintile	1.48 (1.02–2.17)		1.16 (0.76–1.76)	1.14 (0.75–1.74)	1.15 (0.76–1.74)
Fourth quintile	1.07 (0.73–1.58)		0.79 (0.51–1.22)	0.78 (0.50–1.20)	0.78 (0.51–1.20)
Firth quintile (high income)	1.78 (1.23–2.58)		1.25 (0.81–1.94)	1.23 (0.79–1.90)	1.23 (0.80–1.91)
*p*	0.001		0.045	0.050	0.049
*Social support* *^a^*	1.14 (1.03–1.28)			1.12 (1.01–1.25)	1.13 (1.00–1.27)
*p*	0.014			0.040	0.057
*Mental health* *^a^*	1.90 (0.64–5.62)				0.92 (0.27–3.12)
*p*	0.246				0.894

Note: ^a^ Higher mental health and social support scores indicate better mental health and more social support, respectively. Data are from Household Income and Labour Dynamics in Australia (HILDA) survey (2001–2010).

**Table 3:** Odds ratios (95% CI) for the effect of lone-motherhood, socioeconomic status, social support, and mental health on the probability of relapse (n = 3,242).

Covariates	Crude OR	Adjusted OR	Adjusted OR	Adjusted OR	Adjusted OR
*Lone/partnered mother*					
Lone mother	2.64 (1.91–3.65)	2.72 (1.95–3.79)	2.46 (1.69–3.57)	2.36 (1.61–3.46)	2.27 (1.55–3.33)
Partnered mother	1.00	1.00	1.00	1.00	1.00
*p*	<0.001	<0.001	<0.001	<0.001	<0.001
*Age*					
Under 25	1.00	1.00	1.00	1.00	1.00
25–39	0.47 (0.27–0.82)	0.54 (0.31–0.93)	0.58 (0.33–1.02)	0.60 (0.34–1.06)	0.60 (0.34–1.06)
40–54	0.25 (0.14–0.43)	0.29 (0.16–0.50)	0.33 (0.18–0.58)	0.34 (0.19–0.60)	0.34 (0.19–0.61)
55+	0.15 (0.08–0.30)	0.15 (0.08–0.30)	0.18 (0.09–0.36)	0.19 (0.09–0.37)	0.19 (0.09–0.38)
*p*	<0.001	<0.001	<0.001	<0.001	<0.001
*Education*					
High school or less	1.00		1.00	1.00	1.00
Diploma or certificate	0.97 (0.71–1.33)		1.01 (0.73–1.41)	1.01 (0.73–1.41)	1.01 (0.72–1.41)
University degree	0.52 (0.35–0.77)		0.65 (0.42–0.990)	0.66 (0.43–1.01)	0.64 (0.42–0.99)
*p*	0.002		0.0791	0.096	0.085
*Income*					
First quintile (low income)	1.00		1.00	1.00	1.00
Second quintile	0.54 (0.38–0.76)		0.77 (0.54–1.10)	0.78 (0.54–1.12)	0.78 (0.54–1.13)
Third quintile	0.47 (0.32–0.68)		0.78 (0.52–1.18)	0.79 (0.53–1.19)	0.79 (0.52–1.20)
Fourth quintile	0.43 (0.29–0.63)		0.79 (0.52–1.21)	0.80 (0.52–1.22)	0.79 (0.52–1.22)
Firth quintile (high income)	0.44 (0.29–0.65)		0.94 (0.60–1.48)	0.96 (0.61–1.51)	0.96 (0.61–1.52)
*p*	<0.001		0.541	0.557	0.572
*Social support* *^a^*	0.84 (0.73–0.96)			0.92 (0.78–1.07)	0.99 (0.82–1.18)
*p*	0.012			0.277	0.892
*Mental health* *^a^*	0.09 (0.03–0.30)				0.21 (0.05–0.96)
*p*	<0.001				0.044

Note: ^a^ Higher mental health and social support scores indicate better mental health and more social support, respectively. Data are from Household Income and Labour Dynamics in Australia (HILDA) survey (2001–2010).

## 4. Discussion

This study found that, compared to partnered mothers, lone mothers are less likely to quit smoking and among those who quit, they are more likely to relapse. The fact that lone mothers have lower socioeconomic status, receive less social support, and report worse mental health, explains part of the association of lone motherhood with smoking cessation and relapse, but a large part of the association remains unexplained. 

These findings are consistent with previous studies on the relationship between lone motherhood and smoking prevalence. Siahpush *et al*. [3] and Siahpush [2] had shown that in Australia, the high smoking prevalence among lone mothers was partly explained by having low levels of education, living in rental housing, receiving government benefits, residing in disadvantaged neighborhoods, living with no other adult, having no partner, or reporting compromised mental health. However, our findings are not consistent with a British study which used a sample of women aged 25–34 years who were recruited via patient lists of physicians [9]. This study reported no difference in quitting rates between lone mothers and other women after controlling for father’s occupation, age of leaving full-time education, cohabitation status, and age of entry into motherhood [9] Our findings were also inconsistent with another British study which used a cross-sectional design and a sample of women aged 33 years and found no “lone mother effect” on smoking prevalence after controlling for socioeconomic factors [38]. The present and previous studies [2,3] indicate that in Australia, there is an independent effect of lone motherhood on smoking. More research is needed to explore the factors that account for this effect. Two sets of factors, which were not available in the dataset we employed, need to be considered. The first set includes the common predictors of smoking cessation and relapse such as intention to quit, nicotine dependency, self-efficacy to quit and stay abstinent, having many friends who smoke, age of smoking initiation, having smoking restrictions at home, psychological factors, social participation, and psychosocial variables [17,39,40,41]. The second set includes factors that are related to motherhood such as age of entry into motherhood and factors that may be unique to the lives of lone mothers such as experiencing boredom from the monotonous routine of the day (among lone mothers who do not work outside the home), experiencing social stigma, and being too preoccupied with motherhood responsibilities or relationship changes and uncertainties to find enough time to plan for quitting smoking [3].

Consistent with many previous studies that show an association of socioeconomic status with smoking behavior [15,16,17], we found that smokers with lower education were less likely to quit and more likely to relapse. Our study showed that social support was not a predictor of either smoking cessation or relapse. Previous findings on this relationship are mixed [18,19,20,21,42,43]. While the 2000 clinical practice guidelines, Treating Tobacco Use and Dependence [44], recommended that smoking cessation programs should provide and promote social support for smokers who are trying to quit, the most recent updated guidelines excluded this recommendation [45]. This reversal was a result of the emerging literature questioning the benefit of using a smoker’s social network to facilitate cessation [42,43]. Our finding that lone mothers with lower mental health scores were more likely to relapse was consistent with previous studies that have reported an association between poor mental health, more severe withdrawal symptoms [26], and a higher likelihood of relapse [46]. 

While the current and other studies [2,3] have conceptualized socioeconomic status, social support, and mental health as mediating the relationship between lone motherhood and smoking behavior, it is possible that the direction of causality is different from what has been implied here. For example, it is conceivable that a woman’s low socioeconomic status or mental health problems would lead to circumstances that affect lone-motherhood. In such cases these variables would be more distal to smoking and would influence it through lone-motherhood.

A major strength of the study was the nationally representative and longitudinal nature of the data. Instead of retrospectively assessing smoking cessation or relapse, we were able to follow smokers and ex-smokers and record their smoking behavior at a subsequent follow-up time. Furthermore, by pooling data from ten survey waves, we were able to conduct analyses on a relatively large number of lone and partnered mothers. A major weakness was the measurement of smoking which was self-reported. However, self-reported smoking status in surveys of the general population has been validated with cotinine [47] and the amount of misclassification (*i.e.*, proportion of self-reported non-smokers with increased cotinine levels indicative of active smoking) is very low (for example, 0.9% [48] and 1.4% [49]) in most community-based studies [50]. Another weakness of the study, as is the case with most longitudinal population surveys, is non-participation of a segment of the target sample and attrition. In general, individuals of a lower socioeconomic background are less likely to participate in surveys [51]. However, there is little evidence that survey non-participation results in biased study findings [51,52,53]. Although there are mixed findings about socioeconomic variations in attrition in longitudinal surveys [54], attrition in HILDA has been found to be highest among individuals who are unemployed or work in low skilled occupations [55]. While we cannot estimate the effects of non-participation or attrition on the results of the study, we believe that by including socioeconomic indicators as covariates in the regression models, we adjust for these effects to some extent. While employment status and occupation were not included in the models, in analyses not reported here we found that they were not related to cessation or relapse and thus non-participation or attrition base on these variables is not likely to bias the results of the study. While efforts to reduce the smoking prevalence among lone mothers should focus on their socioeconomic position, availability of social support, and addressing mental health issues, other factors unique to the lives of lone mothers also need to be taken into account. More research needs to be conducted to find and address other factors that can explain the association of lone-motherhood and smoking behavior.

## 5. Conclusions

Smoking among lone mothers is an important public health issue not only because it has deleterious health effects but also because it affects the children of lone mothers. Smoking by lone parents has been consistently associated with smoking initiation among children [56,57,58]. Furthermore, exposure to second-hand smoke among children is the cause of many serious health consequences such as infant death syndrome, acute respiratory infections, and ear problems [59]. Thus, any effort to reduce smoking among lone mothers will undoubtedly have a beneficial effect on their children’s health. 

In Australia, over one third of socio-economic disparities in mortality are due to the differences in smoking prevalence across socioeconomic groups [60]. While similar data does not exist to compare the general population with the highly disadvantaged groups such as those who have experienced lone motherhood, we can expect lone motherhood to be at least as important as socioeconomic status in determine health disparities. This is because the prevalence of smoking is astonishingly higher in this group than the general population [3]. Thus, developing smoking cessation programs that are effective among lone mothers can contribute to the amelioration of health disparities. 
